# Small-scale commercial chicken production: A risky business for farmers in the Mekong Delta of Vietnam

**DOI:** 10.1016/j.prevetmed.2021.105470

**Published:** 2021-10

**Authors:** Dinh Bao Truong, Nguyen Van Cuong, Phu Hoang Doan, Nguyen Thi Thuy Dung, Bach Tuan Kiet, Jonathan Rushton, Juan Carrique-Mas

**Affiliations:** a Oxford University Clinical Research Unit, Ho Chi Minh, Vietnam; b Faculty of Animal Science and Veterinary Medicine, Nong Lam University, HCMC, Vietnam; c Sub Department of Animal Health and Production, Cao Lanh, Vietnam; d Centre for Tropical Medicine and Global Health, Nuffield Department of Medicine, Oxford University, Oxford, United Kingdom; e Institute of Infection, Veterinary and Ecological Sciences, University of Liverpool, Liverpool, United Kingdom

**Keywords:** Economic assessment, Chicken, Poultry production, Profitability, Antimicrobials, Vietnam, Production cost, Return on investment

## Abstract

Small-scale farming of meat chicken flocks using local native breeds contributes to the economy of many rural livelihoods in Vietnam and many other low- and middle-income countries (LMICs). These systems are also the target of high levels of antimicrobial use (AMU); however, little is known about the profitability and sustainability of such systems. Since small-scale farms are commercial enterprises, this knowledge is essential to develop successful strategies to curb excessive AMU. Using longitudinal data from 203 small-scale (100−2,000 heads) native chicken flocks raised in 102 randomly selected farms in Dong Thap province (Mekong Delta, Vietnam), we investigated the financial and economic parameters of such systems and the main constraints to their sustainability. Feed accounted for the largest financial cost (flock median 49.5 % [Inter-quartile range (IQR) 41.5−61.8 %]) of total costs, followed by day-old-chicks (DOCs) (median 30.3 % [IQR 23.2−38.4 %]), non-antimicrobial health-supporting products (median 7.1 % [IQR 4.7−10.5 %]), vaccines (median 3.1 % [IQR 2.2−4.8 %]), equipment (median 1.9 % [IQR 0.0−4.9 %]) and antimicrobials (median 1.9 % [IQR 0.7−3.6 %]). Excluding labor costs, farmers achieved a positive return on investment (ROI) from 120 (59.1 %) flocks, the remainder generating a loss (median ROI 124 % [IQR 36–206 %]). Higher ROI was associated with higher flock size and low mortality. There was no statistical association between use of medicated feed and flock mortality or chicken bodyweight. The median daily income per person dedicated to raising chickens was 202,100 VND, lower than alternative rural labor activities in the Mekong Delta. In a large proportion of farms (33.4 %), farmers decided to stop raising chickens after completing one cycle. Farmers who dropped off chicken production purchased more expensive feed (in 1000 VND per kg): 11.1 [10.6−11.5] vs. 10.8 [10.4−11.3] for farms that continued production (p = 0.039), and experienced higher chicken mortality (28.5 % [12.0−79.0 %] vs. 16 [7.5−33.0 %]; p = 0.004). The rapid turnover of farmers raising chickens in such systems represents a challenge to the uptake of messages on appropriate AMU and chicken health. To ensure sustainability of small-scale commercial systems, advisory services need to be available to farmers as they initiate new flocks, and support them in the early stages to help overcome their limited experience and skills. This targeted approach would support profitability whilst reducing risk of emergence of AMR and infectious disease from these systems.

## 1 Introduction

In Vietnam and many other low- and middle-income countries (LMICs) raising small-scale chicken flocks is a common activity that contributes to the income of many rural households. In addition to providing food security and income, such farming systems help promote community relations (Alders and Pym, 2009). The Mekong Delta region of Vietnam (human population 21.5 million in 2019) represents ∼13 % of the total national chicken meat output (840,000 tons in 2018) (General Statistics Office, 2018a). Chicken production in the area is predominantly semi-intensive (including backyard and small-scale), and is typically based on slow-growing native breeds (FAO, 2008; Lan Phuong et al., 2015). Production is however hampered by a high incidence of parasitic (Van et al., 2020a,b), viral and bacterial diseases (Van et al., 2020a,b), often resulting in high mortality losses (Carrique-Mas et al., 2019b). Furthermore, levels of AMU in these systems are particularly high. A recent survey in the area reported that, on average, farmers administered 323.4 (Standard Error of the Mean ±11.3) mg of antimicrobial active ingredients (AAI) per kg of chicken sold (Cuong et al., 2019). In addition, chickens are often raised on commercial medicated feed (estimated to amount to ∼85 mg/kg chicken sold) (Cuong et al., 2021). A total of 42 different antimicrobials, many of which are of critical importance by the World Health Organization (WHO) are used by chicken flocks in the area (Cuong et al., 2019). Poultry farmers often use antimicrobials with the aim of preventing disease, especially during the brooding period, since antimicrobials are viewed as a cheaper alternative than other disease control measures (Truong et al., 2019). Antimicrobials are typically sold over the counter and prices are generally very low (estimated in ∼0.40 cents of 1 USD per daily dose administered to a 1 kg chicken) (Dung et al., 2020). The high amounts of antimicrobials used, often for preventive purposes, is of concern given the high levels of resistance among pathogens (including foodborne pathogens) and commensal bacteria in chicken production systems in the area. Economic analyses have been performed for broiler production systems in Pakistan and Indonesia (Afzal and Khan, 2017; Coyne et al., 2020b) or village chicken production systems in Myanmar (Henning et al., 2013, 2007). However, there are limited published data quantifying the financial flows within small-scale farms raising native chickens that are so common in Vietnam and other Southeast Asian countries. These farming units are typically smaller than their counterpart broiler farms, feed/water dispensation is manual, and birds are always raised at ambient temperatures. A main challenge for studying these systems is their often lack of record-keeping practices (Coyne et al., 2019). Using economic, disease and production data from cohorts of small-scale chicken flocks raised in the Mekong Delta over 18 months period, followed from day-old to slaughter age, we characterized the cost structure of such systems with the aim of quantifying the fraction spent on antimicrobials and other key inputs. The integration of data on feed medication and AMU allowed us to investigate the impact of these parameters on enterprise productivity. An understanding of the economic parameters that underpin small-scale production systems is a pre-requisite for developing and implementing strategies to improve animal health, whilst reducing excessive AMU in Vietnam and elsewhere in Southeast Asia.

## 2 Materials and methods

### 2.1 Study area and data collection

The study was conducted in Cao Lanh and Thap Muoi districts, Dong Thap province, (Mekong Delta, Vietnam) from October 2016 to March 2018. The human population of the province (2017) was 1.69 million. The population densities are 500 people/km^2^ (General Statistics Office, 2018b) and 127 chicken/km^2^ (Sub-Department of Animal Health of Dong Thap, 2017). The study was based on data collected during the baseline phase of an intervention study conducted over 18 month period (Carrique-Mas and Rushton, 2017). We aimed to recruit 120 representative farms raising chicken flocks with more than 100 heads each, managed as all-in-all-out (i.e. single age), raising chicken more than 50 % of the time in the past. The farm selection was based on the census of household-raising livestock held by the veterinary authorities.

Enrolled farmers that consented to the study were provided with diaries and were trained to weekly record information on source of day-old chicks (DOCs), disease, mortality, as well as types and amounts of feed used and health-related products (antimicrobials, vaccines, antiparasitic drugs, disinfectants), and any equipment purchased. A small stipend was given to farmers to compensate them for the time spent on data collection. Data on all costs incurred by farmers to raise the flocks, the weight of chickens at point of sale and the income generated from chicken sales were also recorded. Farms were visited four times over each production cycle by a small dedicated team of field staff (all qualified veterinarians) to review the information collected by farmers. The field staff were trained by a project co-ordinator to collect the data in a consistent way. The project co-ordinator often visited the farms alongside field staff. The data were transferred onto a questionnaire and were then uploaded to a central database using a web application for further analysis. This study was granted ethics approval by the Oxford Tropical Research Ethics Committee (OxtTREC) (Ref. 5121/16) and by the local authorities (People’s Committee of Dong Thap province).

### 2.2 Data analyses

The sum of all financial costs incurred in procuring the DOCs and raising the flocks until slaughter age were computed as input data; output data consisted of the revenues derived from the sale of chickens. For each flock we computed the difference between inputs and outputs, excluding the costs of labor. Labor costs were analyzed separately, since they are an opportunity cost, not a financial one. Antimicrobial in feed was included in feed cost, since in these settings farmers do not add antimicrobials to the feed themselves. Instead, they typically administered them mixed in drinking water. The feed cost (with or without antimicrobials) were considered separate from the expense on antimicrobials given in water. We calculated the return on investment value without labor costs (ROI) for each flock raised (Eq. 1) (Zamfir et al., 2016).

(1) $$ROI=\frac{Revenue-Total\;Cost}{Total\;Cost}*100$$

The resulting values were interpreted as <0, negative ROI value and negative profit; 0 to ≤100, positive ROI value and negative profit (i.e. the investment of 1 Viet Nam Dong (VND) gave a return ≤1 VND); >100, positive ROI value and positive profit (i.e. investment of 1 VND gave a return of ≥1 VND). Financial costs and revenues were expressed ‘per chicken sold’ at the end of each production cycle.

Farm- and flock-related factors influencing the ROI excluding labor for each flock produced were investigated by building a linear mixed random-effects model with the continuous variable ROI as an outcome. The ROI values were square root-transformed in order to improve their distribution’s normality for subsequent modelling. Farm-related variables included district location, owner’s gender, owner’s age, number of staff (including the owner), experience in commercial poultry farming (in years) and education achievement of the owner. Flock-related variables were flock size (No. chickens purchased), duration of the production cycle (in weeks), number of sources of DOCs and their cost, type of feed (commercial, locally-sourced) and feed price (commercial feed) (per kg), percent of weeks consuming commercial medicated feed, average number of daily doses of antimicrobial administered to 1 kg of live chicken per 1000 kg chicken-days (ADD_kg_ per 1000 kg chicken-days) (Phu et al., 2021), number of antimicrobial-containing products used, number of vaccines (pathogens) per flock, flock cumulative mortality over the production cycle (as percent of chickens purchased), cumulative mortality from week 9 (as percent of chickens purchased), flocks in farms raising more than one flock simultaneously, flocks in farms also raising non-chicken species, and flocks where farmer purchased new equipment. All variables were tested as fixed effects, with ‘farm’ identity included as a random effect. Factors that were significant (p < 0.20) in the univariable analysis were added to the multivariable model in a backward step-wise fashion. We tested the interactions between all remaining significant (*p* < 0.05) variables. The package “lme4″ in R (Bates et al., 2015) was used.

We investigated the correlation between all modelled variables (including weight of chickens at sale) using the Kendall’s rank test. Of particular interest was the association between (1) price of DOCs and (i) cumulative mortality, (ii) duration of the production cycle and (iii) chicken weight at sale; (2) No. of ADD_kg_ (per 1000 kg chicken-days) and (i) cumulative mortality or (ii) flock size and (iii) chicken weight at sale; (3) percent of weeks on medicated feed and (i) cumulative mortality, (ii) flock size and (iii) chicken weight at sale; and (4) cumulative mortality and flock size.

We related the income generated from the flocks to the total time spent by the farm owner and other staff (including relatives) tending the flocks (Eq. 2).

(2) $$Daily\;income=\frac{Total\;revenue-Total\;cost}{Working\;time\;\left(days\right)*Number\;of\;workers}$$

$$Working\;time\;\left(days\right)=\frac{No.hours\;per\;day}{8\;hours}*7\;days\;*Duration\;of\;cycle\;(weeks)$$

All financial revenues and costs were expressed in thousands (1000 s) of Vietnam Dong (VND). We investigated the differences between flocks that were and were not followed by a subsequent one within 8 months (i.e. continued/discontinued production) with respect to variables included in the previous analyses. The chosen criterion ensured that seasonal farmers (i.e. those that regularly raise only one cycle per year, typically for the annual Tet holiday) were not considered discontinued production. Pearson’s Chi-squared tests were used for proportions and (non-parametric) Wilcoxon rank sum tests were used for continuous data.

## 3 Results

### 3.1 Description of study flocks

A total of 207 chicken farmers registered in the official census (2014) were invited to an introductory meeting where the aims of the project were explained; 199 attended the meeting. A total of 88 restocked with flocks of ≥100 chickens within the following six months. Additionally, we recruited 14 farms from information provided by veterinary drug shops and commune animal health workers in the area. Therefore, a cohort of 102 farms were finally enrolled. Each farm was observed at least for one cycle. A total of 203 flock production cycles were investigated and their full description can be found in Table 1. Cumulative mortality reached 100 % in 8 (3.9 %) flocks that were affected by an outbreak of severe disease. The main financial revenue from raising these flocks derived from the sale of live chickens for meat. In all cases farmers collected manure (used litter) and feathers and were used to fertilize crops with negligible contribution to farmers’ income.

**Table 1:** Description of key variables related to small-scale commercial chicken flocks raised in Dong Thap province (Mekong Delta, Vietnam).

	Variable and level	Median [IQR] or Number (%)	Range (Min.-max.)
Farms (n = 102)	Male farm owner	89 (88.1 %)	
	Farm owner’s age (years)	46 [38−55]	24−72
	No. of staff (incl. owner)	2 [1−2]	1−4
	Experience in commercial poultry farming (years)	2 [2−3]	0−10
	Education achievement of owner (%)		
	*Primary school*	26 (25.7 %)	
	*Secondary school*	42 (41.6 %)	
	*High school*	28 (27.7 %)	
	*University or higher*	5 (5.0 %)	
Flocks (n = 203)	Flock size	300 [201−502]	100−1,530
	Duration of cycle (weeks)	18 [16−20]	7−29
	Price of DOC (unit) (in 1000 s VND)	10.0 [8.7−11.7]	4.8−23.7
	Feed type		
	*Commercial feed only*	52 (25.6 %)	
	*Commercial feed & locally-sourced feed*	151 (74.4 %)	
	Price of commercial feed (per kg) (in 1,000′s VND)	10.9 [10.4−11.3]	9.2−34.9
	Percent weeks on commercial medicated feed	55.6 [25.0−100.0]	0.0−100.0
	Average No. ADD_kg_ (per 100 chicken-days)	27.4 [13.3−53.4]	0.0−176.7
	No. vaccines (pathogens) per flock	4 [3−4]	1−7
	Cumulate mortality over whole cycle (%)	18 [8−40]	0−100
	Cumulative mortality from week 9 (%)	2.5 [0−15.1]	0−100
	>1 flock raised in the farm at the same time	136 (66.9 %)	
	Flock raised on farms with non-chicken species*	180 (88.7 %)	
	Purchased new equipment	148 (72.9 %)	
	Bodyweight of chickens at sale (kg)	1.5 [1.4−1.7]	1.0−2.9

* Including ducks, Muscovy ducks, quails, pigs, goats and cattle.

### 3.2 Cost structure over the flock production cycle

**Fig. 1:**
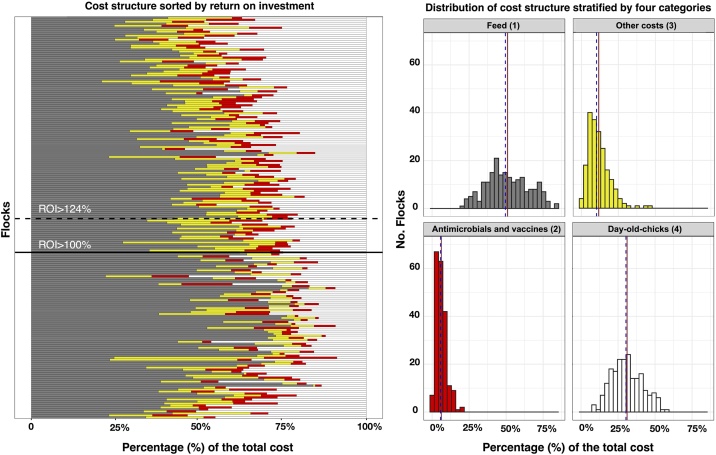
Graphic representation of the cost structure of small-scale chicken flocks raised in Dong Thap province, sorted by return on investment (ROI) (left), and stratified by four categories: (1) Feed; (2) Antimicrobials and vaccines; (3) Other costs; (4) Day-old chicks (Right). Flocks that make a positive profit have ROI > 0. Solid line (mean); dashed line (median).

The financial cost across flocks (in 1000 s VND) incurred in raising one chicken from day-old to slaughter, percentage of the components in cost (feed cost, DOCs, non-antimicrobial health supporting products, vaccine, antimicrobial, other costs) and the revenue obtained per chicken sold is displayed in Table 2. The median revenue obtained per kg chicken sold was 70.0 thousand VND [Inter-quartile range (IQR) 65.0–76.9]. All flocks were raised on commercial feed containing antimicrobials over a median of 55.6 % weeks [IQR 25.0−100.0]. The mean price (by product) of commercial medicated feed was estimated in 11.2 (Standard Deviation (SD)) ±2.5 1000 s VND/kg, statistically higher than the price of non-commercial feed (11.1 SD ± 0.4 per kg) (Welch’s *t*-test 2.19, *p* = 0.03). A total of eight antimicrobial active ingredients (AAIs) were identified from the examination of the labels of these commercial feed formulations (avilamycin, bacitracin, chlortetracycline, colistin, enramycin, flavomycin, oxytetracycline, virginamycin). However only three AAIs, chlortetracycline, bacitracin and enramycin, amounted to >90 % of total use (data not shown). Forty-one-point one percent (41.1 %) of chicken feeds in study area contain antimicrobials. Feed contained a variety of antimicrobials in different concentrations. The median ROI across all flocks was 124 % [IQR 36–206 %]; therefore, for each VND invested there was a return of VND 1.24. For 120/203 (59.1 %) flocks, farmers obtained a positive profit (ROI>100 %), whereas for 83 (40.9 %) farmers obtained negative profit (ROI<100 %) (i.e. financial losses). The main cost categories sorted by flock ROI by flock are presented in Fig. 1. Generally, higher ROI was associated with a smaller fraction of feed costs.

**Table 2:** Financial costs and revenue obtained per chicken unit.

**Item**	**Median** **(**x 1000 s VND)	**IQR** **(**x 1000 s VND)	**Median (%)**	**IQR (%)**
**Financial cost**	**48.3**	**36.8 – 78.0**		
+ Feed cost (commercial and locally –source feed)			49.5	41.5−61.8
+ DOCs			30.3	23.2−38.4
+ Non-antimicrobial health supporting products (vitamins, anti-parasitic drugs)			7.1	4.7−10.5
+ Vaccines			3.1	2.2−4.8
+ Antimicrobial			1.9	0.7−3.6
+ Other costs (equipment, litter, electricity and disinfectants)			1.9	0.0−4.9
**Revenue**	**108.7**	**99.4−121.7**

**Fig. 2:**
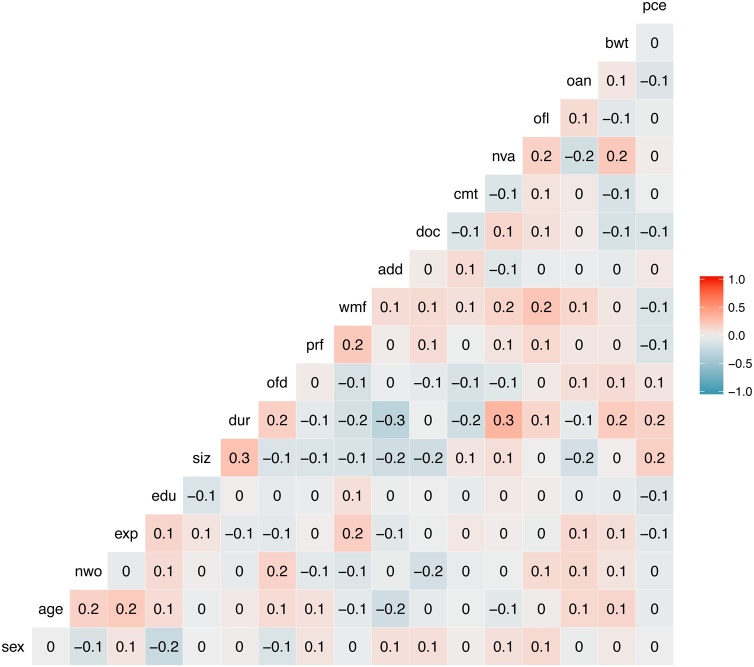
Correlation matrix of study variables. *sex*: owner’s gender, *age:* owner’s age; *nwo:* number of workers; *exp:* experience in poultry farming (years); *siz:* flock size; *dur:* length of cycle; *ofd:* usage locally-sourced feed; *prf:* feed price; *wmf:* percent weeks on medicated feed; *add:* average weekly antimicrobial daily dose; *doc:* price DOCs; *cmt:* flock cycle cumulative mortality; *nva:* number of pathogens vaccinated; *ofl:* other flock raised in farm; *oan:* other animal raised in farm; *bwt:* average weight of chicken; *pce:* purchase of new equipment.

The correlation between all modelled variables (including weight of chickens at sale, not modelled) is displayed in Fig. 2. There was no association between the cost of DOCs (per unit purchased) and cumulative mortality (Kendall’s rank correlation *tau*=-0.08, *p* = 0.09) or the duration of the flock cycle (*tau*=-0.01; *p* = 0.84) or chicken weight at sale (*tau*=-0.03, *p* = 0.53). There was a small, significant negative correlation between the total number of ADD_kg_ administered and flock size (*tau*=-0.14, *p* = 0.003). There was no association between the total number of ADD_kg_ administered and cumulative mortality (*tau* = 0.05, *p* = 0.29) or chicken weigh at sale (*tau* = -0.05, *p* = 0.27). No association was found between percent of weeks on medicated feed and cumulative mortality (*tau* = 0.04, p = 0.33) or flock size (*tau* = -0.08, *p* = 0.09) or chicken weight at sale (*tau* = 0.04, *p* = 0.35). There was no correlation between cumulative mortality and flock size (*tau* = 0.07, *p* = 0.14).

### 3.3 Factors associated with ROI excluding labor costs

Only two factors were independently associated with ROI, flock size (*β* = 0.08, *p* < 0.001), and cumulative mortality (*β*=-1.45, *p* < 0.001) (inverse association) (Table 3). As expected, the average weight of chickens sold was strongly associated with flock ROI (*β* = 0.51, *p* = 0.001), and therefore was not included in the multivariable model. The predicted outcomes for different values of the two significant variables are given in Supplementary Material 1. Mortality was the single most influential driver of ROI.

**Table 3:** Linear mixed models investigating factors associated with ROI of raising small-scale meat chicken flocks.

Variable and level	Univariable			Multivariable*		
	*B*	SE	*p*-value	*β*	SE	*p*-value
Female farm owner	0.04	0.09	0.764			
Farm owner’s age (years)	0.004	<0.01	0.211			
No. of staff (incl. owner)	0.04	0.07	0.514			
Experience in commercial poultry farming (years)	0.02	0.02	0.227			
Education achievement (baseline = Primary school)						
*Secondary school*	−0.01	0.11	0.918			
*High school*	−0.23	0.12	0.054			
*University or higher*	−0.15	0.21	0.477			
Flock size (per 100 chickens)	0.07	0.01	<0.001	0.08	0.008	<0.001
Duration of cycle (month)	0.21	0.04	<0.001	0.04	0.02	0.071
Price of DOC unit (in 1000,000 s of VND)	−0.03	16.3	0.998			
Use of locally-sourced feed	0.18	0.06	0.052	0.03	0.04	0.408
Price of commercial feed (per kg) (in 1000 s of VND)	−0.01	0.01	0.588			
Percent weeks on commercial medicated feed	−0.12	0.11	0.254			
Average No. ADD_kg_ (per100 chicken-days) (square root)	−0.002	<0.01	0.032	−0.001	<0.001	0.244
Flock vaccinated against >4 pathogens	0.08	0.09	0.33			
Cumulative mortality over whole cycle (%)	−1.42	0.07	<0.001	−1.45	0.06	<0.001
>1 flock raised in the farm at the same time	−0.11	0.07	0.162	0.01	0.03	0.659
Flock raised on farms with non-chicken species	−0.02	0.12	0.884			
Purchased new equipment	0.06	0.07	0.428			

* Multivariable model intercept = 1.31, Standard Error (SE) ±0.12; Value of square root – transformed ROI: median 1.49 [IQR.1.16–1.75]

### 3.4 Income generated per day of labor

**Fig. 3:**
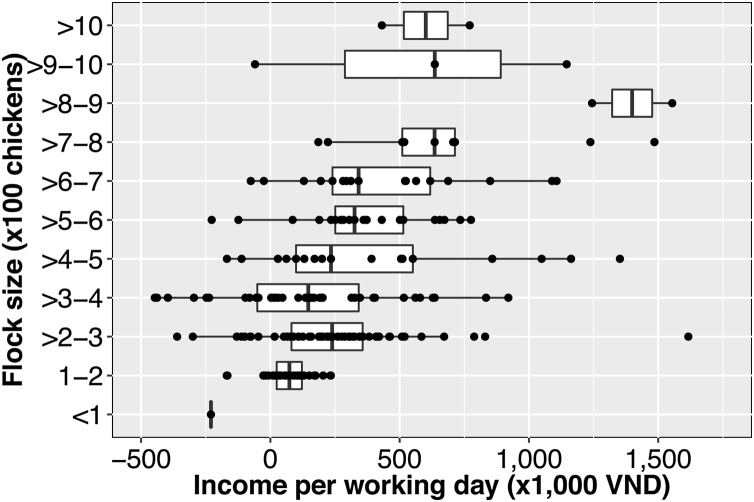
Income generated per day of labor according to flock size.

**Fig. 4:**
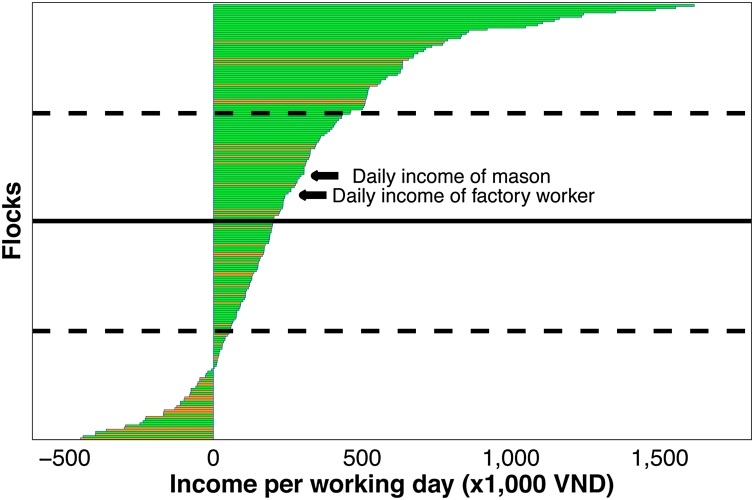
Distribution of income generated per day of labor among flocks. Solid line (mean); dashed lines (1st and 3rd quartile); green (farmers that continued production); orange (farmers that discontinued production).

A median of 399 [IQR 266–613] person-hours (equivalent to 49.8 [IQR 33.3–76.5] working days were employed to raise one flock, and a median of 2.3 [IQR 1.3−4.4] person-hours per chicken raised. The median daily income per person in chicken production was 202.1 thousands of VND [IQR 56.5–461.0]. However, for 33 (16.2 %) flocks, farmers labor income was negative. There was a moderate positive correlation between income per working day and flock size (*tau* = 0.29, *p* < 0.001) (Fig. 3 & 4).

### 3.5 Farms that discontinued chicken production

Of 197/203 (97 %) flocks that could be evaluated for the criterion of whether or not farmers continued raising chickens within 8 months after their sale, 46 (33.4 %) were not followed up by a subsequent flock (i. e. discontinued farming). Table 3 shows the differences with regards to all variables investigated. Farmers that did not raise further flocks had purchased feed prices (per kg) at a higher cost compared with flocks in farms that continued production (11.1 [10.6−11.5] 1000 VND vs. 10.8 [10.4−11.3]; p = 0.039). In addition, these farmers that discontinued production also experienced higher mortality in their flocks (28.5 % [12.0−79.0%] vs. 16 % [7.5−33.0%]; *p* = 0.004). As expected, the ROI of flocks for flocks followed up were also higher (53.8 % [-44.6−132.9%] vs. 139.9 % [55.2−238.5%]) (Table 4).

**Table 4:** Description of farm/flocks that discontinued/continued chicken production after one flock.

Variable	Discontinued production (N = 46)		Continued production (N = 151)		*p*-value
	Median [IQR]	No. (%)	Median [IQR]	No. (%)	
Flock size	303 [202−404]		300 [201−507]		0.956^⸶⸶^
Duration of cycle (weeks)	19 [16−21]		18 [16−20]		0.468^⸶⸶^
Price of DOC unit (in 1000 s of VND)	10.0 [8.8−11.7]		10.0 [8.5−11.7]		0.801^⸶⸶^
Feed type					
*Commercial feed only*		9		39	0.502^⸶^
*Commercial feed & locally-sourced feed*		37		112	
Price of commercial feed (per kg) (in 1,000′s VND)	11.1 [10.6−11.5]		10.8 [10.4−11.3]		0.039^⸶⸶^
Percent weeks on medicated feed	50.3 [22.5−100.0]		55.5 [26.2−100.0]		0.651^⸶⸶^
Average No. ADD_kg_ per 1000 chicken-days	329 [211−639]		266 [126−489]		0.074^⸶⸶^
No. vaccines (pathogens) per flock	4 [3−4]		4 [3−5]		0.513^⸶⸶^
Cumulate mortality over whole cycle (%)	28.5 [12.0−79.0]		16.0 [7.5−33.0]		0.004^⸶⸶^
Cumulate mortality from week 9 (%)	6.9 [0.2−58.1]		1.7 [0.0−11.0]		0.008^⸶⸶^
>1 flock raised in the farm at the same time		31 (67.4 %)		102 (67.5 %)	1.0^⸶^
No. flocks raised on farms with non-chicken species		39 (84.8 %)		137 (90.7 %)	0.270^⸶^
Purchased new equipment		40 (87.0 %)		47 (68.9 %)	0.025^⸶^
Bodyweight of chicken at sale (kg)	1.5 [1.4−1.6]		1.5 [1.4−1.7]		0.389^⸶⸶^
ROI (%)	53.8 [-44.6−132.9]		139.9 [55.2−238.5]		<0.001^⸶⸶^

Legend: ^⸶^Result from Pearson’s Chi-squared test for discrete data; ^⸶⸶^Result from Wilcoxon rank sum test for continuous data.

## 4 Discussion

Our study shows that, in the Mekong Delta of Vietnam, raising flocks of 100−2,000 meat chickens is occasionally profitable (median 1.24 VND returned per 1 VND invested). The financial profit generated from these systems (per chicken produced) generally increased with flock size. However, for 40.9 % of flock farmers incurred in financial losses. The main explanatory factors for the losses identified were a small flock size and high mortality. The (high) observed cumulative mortality (median 18 %) is likely to reflect a high incidence of bacterial, viral and parasitic diseases identified in the area (Carrique-Mas et al., 2019b; Van et al., 2020a,b). The median cumulative mortality reached 28 % in flocks raised in farms that discontinued production.

The proportion of costs attributable to DOCs our study was 30.3 %, higher than for Indonesian (25 % costs) and Pakistani (22–29 %) broiler flocks (Afzal and Khan, 2017; Coyne et al., 2020b). This is probably due to the absence of commercial hatcheries in the study province (Dong Thap), with DOCs typically being delivered through a complex network of intermediate traders. However, this may also reflect the fact that feed and others costs in Vietnam were lower than the other Asian countries. We did not attempt to characterize the breed identity of study flocks due to its complexity. The choice of adequate breed lines should however be important in order to maximize production in these small-scale systems.

Our study indicates that commercial feed was still the greatest single most important financial expense incurred to raise chicken flocks (median 49.5 % of all costs). In Pakistani broiler flocks feed represented 58.1–63.6 % depending on farm type (Afzal and Khan, 2017), and in Indonesia ∼70 % (Coyne et al., 2020b). It is possible that some of savings in our study flocks were due to the provision of locally-sourced feed (local plants, rice gain) at no cost. Disinfectants account for a very small fraction in these farms, as in the area they are often provided by the local veterinary authorities free of charge. Our results indicate that, overall, the cost (per kg) of medicated feed was about the same as non-medicated feed (the different in price per kg was about approximately 1%); however, we could not demonstrate any impact of medicated feed (variable named percent of week on medicated feed) on bodyweight and mortality, even though that the final aim of using antimicrobials was to prevent and treat disease, contributing to increased income (Truong et al., 2019). In our study, there was a considerable diversity in feed products with regards to their antimicrobial composition as has been recently shown (Cuong et al., 2021). The evidence of the impact of antimicrobials in feed (generally as antimicrobial growth promoters, AGPs) on animal productivity has generally been mixed and highly variable depending on the studies and production types (Laxminarayan et al., 2015). In our study, more affordable commercial feed, but not the inclusion of AGPs, was associated with the sustainability of this production system. In contrast, the magnitude of AMU in water (expressed as ADD_kg_) was higher in flocks that discontinued production, reflecting a higher incidence of disease and mortality.

In our study, we estimated that medicines accounted for ∼9% of total input costs, compared with 5–6 % in Pakistan and ∼1.3 % in Indonesia. However, in our study the fraction of antimicrobials to total medicine cost was relatively small. In contrast, the expense on vaccines was higher in our study flocks (3.1 %) than in Indonesian (0.8 %) and Pakistani broiler studies (∼1.5) (Afzal and Khan, 2017; Coyne et al., 2020b). Vaccination of flocks against pathogens is a widespread practice in the Mekong Delta, with >85 % flocks vaccinated against three or more pathogens, the most common being, in decreasing order, Newcastle Disease, Highly Pathogenic Avian Influenza, Infectious Bursal Disease, fowl pox, fowl cholera and Infectious Bronchitis (data not shown).

Our results reflect a relatively small impact of AMU on overall financial costs (<2%), in spite of the high volume of antimicrobials used (previously described in detail using a number of metrics (Cuong et al., 2019)). This is reflective of the generally low price of antimicrobials available to Mekong Delta farmers. For example, a daily dose of an average antimicrobial-containing product (per kg of chicken) has been estimated to retail at ∼0.40 cents of 1 US$, depending on product (Carrique-Mas et al., 2019a; Dung et al., 2020). Our findings are not dissimilar to costs of antimicrobials intended for small-scale pig farms in Vietnam (<2% total costs) (Coyne et al., 2020a).

Even though our study is of relatively large scale, we believe its findings are restricted to native chicken farming systems in the Mekong Delta of Vietnam and probably cannot entirely be extrapolated to industrial chicken farming in small-commercial scale farming in other regions of the country. It would have been desirable to obtain a broader understanding of the full economic picture of farming in the area, since in addition to chicken raising, many farms also raised other species (pigs, ducks), as well as growing fruits, fish and rice. However, the access to such detailed data collection is laborious and excessive data collection may jeopardize the commitment of the farmers.

In Vietnam, as well as in other Southeast Asian countries, livestock production systems have become more intensified in recent years in response to increased demand for animal protein (Jabbar, 2015; Soedjana and Priyanti, 2017). The systems described here represent an intermediate type between backyard farming and industrial production. However, increases in flock size presents challenges, such as a higher risk of disease and mortality, as well as higher frequency of AMU, as shown in a recent study (Carrique-Mas et al., 2019b).

Despite the increased popularity of industrial broilers, meat from native breeds is still highly valued by Vietnamese consumers thanks to its distinct taste. Native chicken production still represents a majority of national chicken meat output: of 990,397 tonnes of chicken meat produced in 2019, 43.3 % corresponded to industrial chicken production, the rest corresponding to meat from backyard and small-scale production systems (Anonym, 2019). Native chickens purchased at the farm gate (median price 70.0 thousand VND per kg in our study) reach a much higher retail price than broiler chicken meat purchased at retail (i.e. supermarket) (∼20.0–25.0 thousand VND per kg) (Anonym, 2019).

The estimated daily income from raising chickens in our study (VND 202,120) was considerably lower than that generated from comparable occupations in the rural areas of the Mekong Delta region, such as mason (VND ∼300,000 per day), or factory worker (VND ∼250,000 per day) (authors’ observation) (Fig. 4). A major challenge is the limited available land in this densely populated area and the competing income-generating activities (retail, rice, fruit, duck, pig and fish farming). Most households normally make up their income from a range of activities. Often, several members of the family are engage in raising chickens. For the individual farmer, raising livestock and poultry are often unstable activities due to the seasonality of production, market fluctuations and the regular incursion of infectious diseases. The recent 2019 incursion of African Swine Fever epidemic in Vietnam (Le et al., 2019) resulted in many pig farmers switching to chicken production, with an associated decline in prices of finished chicken. The necessities to undertake multiple occupations are not generally conducive to farmers becoming proficient in chicken husbandry.

### 4.1 Conclusion

We confirmed a high degree of instability in small-scale chicken farming systems in the Mekong Delta, with many farmers ceasing production after one or two cycles. A higher profitability was attained with larger flocks with low mortality. In spite of a high frequency of AMU, expenses on antimicrobials accounted for <2% of total costs. We did not find any impact of medicated feed on overall mortality or productivity in flocks. In order to remain profitable, farm owners need to implement better disease control practices. Achieving this would require official support from the authorities, for example in the form of the provision of advisory services offering short training courses, disease diagnostics, etc. which could be delivered by farmers’ associations and veterinary pharmacists so that farmers can improve their knowledge base on flock health management.

## Declaration of Competing Interest

Non declared.
